# Development of an Alternative In Vitro Rumen Fermentation Prediction Model

**DOI:** 10.3390/ani14020289

**Published:** 2024-01-17

**Authors:** Xinjie Wang, Jianzhao Zhou, Runjie Jiang, Yuxuan Wang, Yonggen Zhang, Renbiao Wu, Xiaohui A, Haitao Du, Jiaxu Tian, Xiaoli Wei, Weizheng Shen

**Affiliations:** 1 College of Electric and Information, Northeast Agricultural University, Harbin 150038, China; wxj921374497@163.com (X.W.);; 2 College of Animal Sciences and Technology, Northeast Agriculture University, Harbin 150038, China; 3 Heilongjiang Academy of Agricultural Sciences Animal Husbandry and Veterinary Branch, Harbin 150086, China; 4 Heilongjiang Dairy Industry Association, Harbin 150040, China

**Keywords:** machine learning, in vitro rumen model, rumen methane, rumen acetic acid, prediction model

## Abstract

**Simple Summary:**

Our objective is to establish an in vitro rumen fermentation model that can dynamically simulate the fermentation process of various total mixed ration (TMR) diets in the rumen of dairy cows, enabling a quantitative investigation of rumen methane and rumen acetic acid concentrations. The models were assessed for their prediction accuracy and precision, while the independent verification experiments confirmed the models’ generalization ability across different total mixed ration (TMR) ratios (C:F). These results show that in vitro rumen models constructed by machine learning methods can be used as a tool to quantify rumen fermentation parameters (methane and acetic acid) and guide the dietary structure optimization of dairy cows.

**Abstract:**

The aim of this study is to identify an alternative approach for simulating the in vitro fermentation and quantifying the production of rumen methane and rumen acetic acid during the rumen fermentation process with different total mixed rations. In this experiment, dietary nutrient compositions (neutral detergent fiber (NDF), acid detergent fiber (ADF), crude protein (CP), and dry matter (DM)) were selected as input parameters to establish three prediction models for rumen fermentation parameters (methane and acetic acid): an artificial neural network model, a genetic algorithm-bp model, and a support vector machine model. The research findings show that the three models had similar simulation results that aligned with the measured data trends (R^2^ ≥ 0.83). Additionally, the root mean square errors (RMSEs) were ≤1.85 mL/g in the rumen methane model and ≤2.248 mmol/L in the rumen acetic acid model. Finally, this study also demonstrates the models’ capacity for generalization through an independent verification experiment, as they effectively predicted outcomes even when significant trial factors were manipulated. These results suggest that machine learning-based in vitro rumen models can serve as a valuable tool for quantifying rumen fermentation parameters, guiding the optimization of dietary structures for dairy cows, rapidly screening methane-reducing feed options, and enhancing feeding efficiency.

## 1. Introduction

Methane, a long-lived greenhouse gas with a relatively extended half-life, exhibits a global warming potential 25 to 30 times higher than that of carbon dioxide. The global annual methane emissions reach 5.35 × 10^8^ t. The majority of methane emissions are produced in ruminant husbandry through enteric fermentation, accounting for 89% of total methane emissions [1,2]. Specifically, bovine methane emissions (excluding buffalo) represent about 75% of the total emissions from ruminants [3]. Methane released by dairy cows represents a significant contribution to greenhouse gas emissions [4]. Therefore, whether from the perspective of environmental protection or animal production, it is extremely necessary to reduce methane emissions from ruminants [5].

Methanogenesis is a fundamental rumen metabolic process, and rumen methane is the product of anaerobic fermentation of structural carbohydrates in ruminant rumen. In animal production, methane emissions can be reduced through using total mixed ration (TMR)nutrition control methods, and the in vitro rumen fermentation technique is a powerful tool in the study of ruminant nutritional regulation [6]. The in vitro method involves simulating the rumen fermentation environment through the use of in vitro enzymes or inoculated rumen microorganisms, and assessing feed fermentation parameters to determine the dry matter degradation rate and metabolizable energy [7,8]. The use of in vitro methods has been widely adopted by scholars to forecast rumen fermentation parameters and assess rumen methane production during rumen fermentation [9,10]. Our previous studies proved that the nutritional value of each TMR diet can be compared intuitively using an in vitro method [11], and we also discovered that the methanogenic potential of different TMR diets can be evaluated using an in vitro gas production method [12]. It can be seen that in vitro methods are a valuable approach for assessing the nutritional value of feed and developing effective nutritional regulation schemes. However, current in vitro fermentation technology has limitations, including its reliance on fistulated animals and complex operational procedures. We also proved the feasibility of using machine learning methods to simulate complex nonlinear problems in dairy production [13,14]. Therefore, our objective is to establish an in vitro rumen fermentation model that can dynamically simulate the fermentation process of different TMRs within the rumen. This model enables us to simulate and quantify rumen fermentation as well as methane production in dairy cows.

Traditional modeling methods, such as the non-linear least squares method, which is commonly used in classic regression algorithms, have found extensive applications across various scientific disciplines. However, their effectiveness is often limited when confronted with intricate biological processes like rumen fermentation [15,16]. These methods often prove inadequate in addressing nonlinearity, high dimensionality, and complex interactions, particularly when dealing with a large number of variables and potential interactions. Moreover, traditional approaches exhibit limitations in simulating the dynamic changes of fermentation processes as they primarily focus on static data analysis rather than predicting system dynamics. Traditional methods heavily rely on the initial selection of the functional form, which can significantly limit their effectiveness. The inadequate choice of an initial function may result in models that fail to accurately capture the intrinsic dynamics of the data. This limitation becomes particularly apparent in intricate biological processes like rumen fermentation, where these methods may not offer reliable insights or predictions [17,18].

This study proposes the use of machine learning techniques to build a predictive model for rumen fermentation parameters. Based on the relevant literature [19,20,21,22,23] and our previous research, we utilize in vitro rumen fermentation data, incorporating factors such as neutral detergent fiber (NDF), acid detergent fiber (ADF), crude protein (CP), and dry matter (DM) to develop the model. The objective is to evaluate and compare the accuracy and precision of various conventional machine learning algorithms in estimating rumen methane and acetic acid production. Additionally, the study aims to demonstrate the model’s ability to generalize across different concentrate-to-forage ratios (C:F) in total mixed rations (TMRs), which is then validated through an independent experiment.

## 2. Materials and Methods

### 2.1. Experimental Design

The necessary sample data for accurately predicting the rumen fermentation parameters of dairy cows were obtained through rigorous feeding and digestion experiments in this study. TMR samples (collected using the four division method) for in vitro fermentation were collected from lactating cows with an average lactation period of approximately 150 days, with a deviation of ±5 days, across various pastures in Harbin, China, in 2018. The TMR was dried at a temperature of 65 °C for a duration of 48 h, and subsequently ground into particles with a size 40 mesh. The fermentation substrate was prepared by measuring and loading 0.2 g of weight into a fiber bag. Each fermentation bag was subject to five repetitions. The neutral detergent fiber (NDF), acid detergent fiber (ADF), crude Protein (CP), and dry matter (DM) contents were determined following the methodology described by Yang et al. [12]. Rumen fluid samples were collected from two Holstein cows weighing approximately 600 kg, using a rumen cannula, two hours after feeding. All collections of rumen fluid from dairy cows were conducted in strict adherence to a standardized pre-feeding protocol, which included providing identical feed and following a consistent feeding regimen. The cows underwent a 14-day period of pre-feeding, and on the 15th day, rumen fluid collection was initiated.

In vitro fermentation was conducted using the method outlined by Weiby et al. [24], with procedures previously reported by Li et al. [25]. The in vitro fermentation experiment was conducted at the Acheng Experimental Base of Northeast Agricultural University. Rumen methane and rumen acetic acid production were measured after a 24 h period of in vitro fermentation. All analyses were performed in quintuplicate, and all data obtained were used for modeling purposes.

### 2.2. Experimental Data and Data Processing

**Figure 1:**
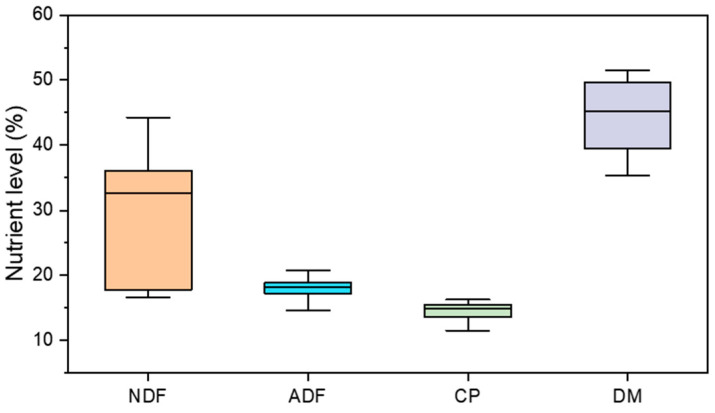
Nutrient levels of the data set (NDF, ADF, CP, and DM).

The dataset consisted of 120 data groups, in which the nutrient levels are depicted in Figure 1. Among these, 90% of the data were randomly selected for training the model, while the remaining 10% were utilized for model testing, as presented in Table 1. To optimize the model parameters, a 10-fold cross-validation approach was employed on the training set.

The calculation formula for normalization of the input data is in Equation (1) (suppose the normalization interval is [*a*, *b*]), and the calculation formula for the inverse normalization of model output can obtained from Equation (2). The process is in reference to [14].

(1) $$T_{i}^{\prime }=a+\left(b-a\right)\cdot \frac{T_{i}-T_{imin}}{T_{imax}-T_{imin}}\left(i=1,2,3,4,5,6\right)$$

(2) $$T_{i}=T_{imin}+(T_{i}^{\prime }-a)\cdot \frac{T_{imax}-T_{imin}}{b-a}\left(i=1,2,3,4,5,6\right)$$

where $T_{i}$ is the input of training sample data, $T_{i}^{\prime }$ is the normalized result, $T_{i}^{\prime }$ ∈ [*a*, *b*], and $T_{imax}$ and $T_{imin}$ are the maximum and minimum values in the data.

### 2.3. Validation Experiment Design

To further validate the robustness and generalizability of the prediction models proposed in this paper, independent testing was conducted using the validation data on a commercial farm in Changchun, China, in 2021. Two total mixed ration (TMR) diets were formulated with compound feed at the Ruminant Nutrition Laboratory of Northeast Agriculture University: T_1_ (concentrate–forage ratio of 40:60) and T_2_ (concentrate–forage ratio of 60:40). Both diets adequately met the nutritional requirements of the animals based on the Cornell–Penn–Miner dairy model (Version 3.08.01). The ingredient composition and chemical analysis are presented in Table 2.

Rumen fluid was collected from three cattle weighing 500 ± 50 kg before the morning feeding time. We conducted the in vitro incubation procedures in accordance with the methods outlined in a previous study [26]. Rumen methane production was measured using a real-time in vitro fermentation system (Qtfxy-6, Jilin Academy of Agricultural Sciences, Jilin, China) as described by Sun et al. [27], and the concentrations of rumen acetic acid were quantitatively determined using gas chromatography techniques. All analyses were performed in triplicate.

### 2.4. Algorithm and Model

In this experiment, three machine learning algorithms, backpropagation (BP), the genetic algorithm (GA)-BP model and support vector machine (SVM), were used to establish the in vitro rumen fermentation models to predict rumen methane and rumen acetic acid, respectively.

#### 2.4.1. BP Model of In Vitro Rumen Fermentation in Dairy Cows

**Figure 2:**
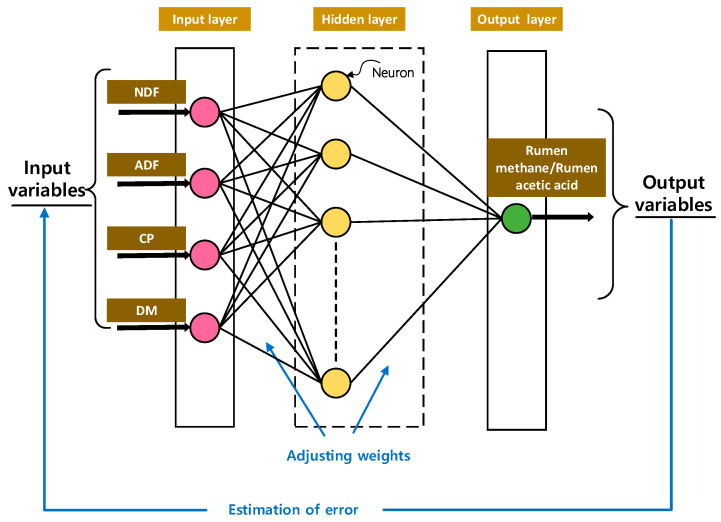
Structure of BP neural network: input variables (NDF, ADF, CP, and DM) and output variable (rumen methane/rumen acetic acid).

The network input layer comprises four input factors, namely NDF, ADF, CP, and DM. The output layer consists of one parameter, either rumen methane or rumen acetic acid. The objective function is defined as Equation (3). The establishment process is in reference to [14], and the structure of the three-layer BP model is shown in Figure 2.

(3) $$Y=F\left(X\right)=f[V\cdot f\left(W\cdot X+\theta_{1}\right)+\theta_{2}]$$

where f() is the single-stage Sigmoid function, X is the input vector; Y is the output value, F(X) is the relationship between the input value and the output value, W is the weight from the input layer to the hidden layer, $\theta_{1}$ is the hidden layer threshold value, V is the weight from the hidden layer to the output layer, and $\theta_{2}$ is the output layer threshold value.

The BP neural network model was implemented using the Python platform in this study, with a total of 3000 training iterations. The network performance test revealed that there were five nodes in the hidden layer.

#### 2.4.2. GA-BP Model of In Vitro Rumen Fermentation in Dairy Cows

The genetic algorithm (GA) is a computational method that draws inspiration from the principles of natural selection and genetic mechanisms found in biological evolution. It is inherently a parallel random search optimization technique designed to solve complex problems by mimicking the process of natural evolution. A key application of GA is in the optimization of weights and thresholds in neural networks [28].

Referring to [29], the procedure for implementing a genetic algorithm begins with the random initialization of a population. This initial population consists of a number of individuals, each representing a potential solution to the problem at hand. The process then involves evaluating the fitness of each individual in the population. The fitness value, defined by Equation (4), measures how well an individual solves the problem or achieves the desired outcome.

The genetic algorithm utilizes a series of operations to evolve the population towards optimal solutions. These operations include selection, crossover, and mutation. The selection process, governed by Equations (5) and (6), determines which individuals are chosen based on their fitness values to pass their genes to the next generation. The crossover operation, described by Equations (7) and (8), involves combining the genetic information of selected individuals to create new offspring. This process allows for the exchange of genetic material between individuals, introducing diversity into the population. The mutation operation, as per Equation (9), introduces random changes to individual genes, further contributing to the diversity and exploration of new solutions.

(4) $$F=\sum_{i=1}^{n}\left|output_{i}-Y_{i}\right|$$

where n is the number of model output nodes, $output_{i}$ is the evaluation value, and $Y_{i}$ is the desired output.

(5) $$f_{i}=\frac{1}{F_{i}}$$

(6) $$p_{i}=\frac{f_{i}}{\sum_{i=1}^{N}f_{i}}$$

where $p_{i}$ is the selection probability of each individual, $f_{i}$ is the inverse of the fitness value, and N is the number of population individuals.

(7) $$a_{sj}=a_{sj}\left(1-b\right)+a_{dj}b$$

(8) $$a_{dj}=a_{dj}\left(1-b\right)+a_{sj}b$$

It can be seen from Equations (7) and (8), the process of crossover occurs between the s-th chromosome and the d-th chromosome at position j, where b∈ (0, 1).

(9) $$a_{kl}=\left\{\begin{array}{c} a_{kl}+\left(a_{kl}-a_{max}\right)\times f\left(g\right),\;r\in \left[0.5,1\right]\\ a_{kl}+\left(a_{min}-a_{kl}\right)\times f\left(g\right),\;r\in [0,0.5] \end{array}\right.$$

where $a_{\max}$ and $a_{min}$ are the upper and lower bounds of genes $a_{kl}$, $f(g)=r_{1}{\left(1-g/G_{max}\right)}^{2}$, $r_{1}\in$ [0, 1], g is the number of iterations, and $G_{max}$ is the maximum number of evolutions.

**Figure 3:**
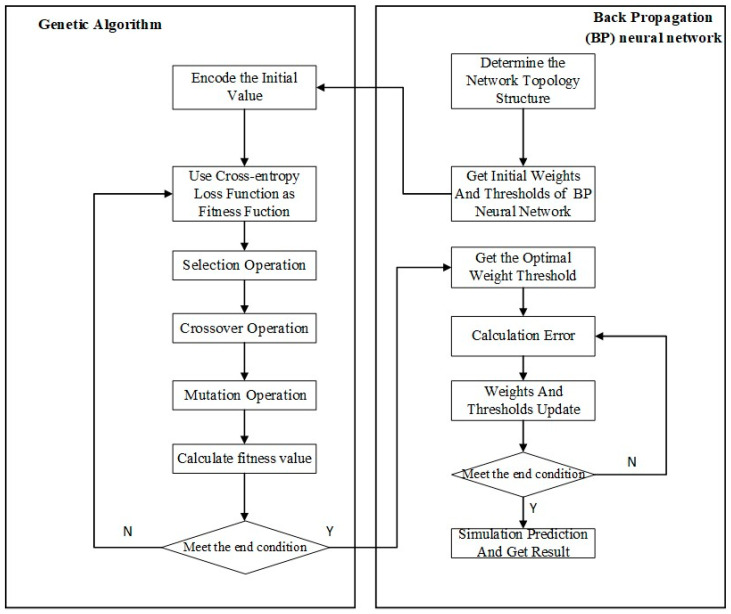
Optimization process of weight and threshold parameters by GA-BP.

Throughout these operations, the genetic algorithm iteratively refines the population. The fittest individuals are more likely to be selected for reproduction, allowing their superior traits to be passed on to successive generations. This iterative process of selection, crossover, and mutation continues over multiple generations until the algorithm converges on an optimal set of parameters. The optimization process and its various stages are visually summarized in Figure 3. 

In essence, the genetic algorithm’s ability to explore a vast search space and its robustness in finding global optima make it an effective tool for neural network optimization and various other complex optimization problems [30].

#### 2.4.3. Support Vector Machine Model of In Vitro Rumen Fermentation in Dairy Cows

Support vector machine (SVM) is a machine learning method with statistics as its theoretical basis. Studies have found that support vector machine is very suitable for the regression of small sample data when dealing with regression problems [13]. For the given training sample, through nonlinear mapping ϕ(x), the low-dimension data are mapped to the high-dimension space, enabling linear regression in this transformed space, as shown in Equation (10).

(10) f(x)=ωϕ(x)+b

where ω is the weight vector, and b is the partial value. The values of ω and b are determined by minimizing the regression risk.

The SVM model in this study is established following the approach described by Shen et al. [14]. In this study, the radial basis kernel function is defined as the kernel functions.

### 2.5. Model Evaluation and Validation Method

The model evaluation method employs the mean absolute error (MAE), mean absolute percent error (MAPE), root mean square relative error (RMSE), and correlation coefficient (R^2) for assessment purposes, which are commonly used in all kinds of predictive performance evaluations [31]. The formulas are as follows:

(11) $$MAE=\frac{1}{n}\sum_{i=1}^{n}\left|Y_{test}^{i}-{\hat{Y}}_{test}^{i}\right|$$

(12) $$MAPE=\frac{100}{n}\times \sum_{i=1}^{n}\left|\frac{Y_{test}^{i}-{\hat{Y}}_{test}^{i}}{Y_{test}^{i}}\right|$$

(13) $$RMSE=\sqrt{\frac{1}{n}\sum_{i=1}^{n}(Y_{test}^{i}-{\hat{Y}}_{test}^{i}{)}^{2}}$$

(14) $$R^{2}=\frac{\sum_{i=1}^{n}(Y_{\mathrm{test}}^{i}-{\hat{Y}}_{\mathrm{test}}^{i}{)}^{2}}{\sum_{i=1}^{n}(Y_{test}^{i}-\bar{Y}{)}^{2}}$$

where n is the total number of test samples, $Y_{test}^{i}$ is the measured value, ${\hat{Y}}_{test}^{i}$ is the simulation value, and $\bar{Y}$ is the average value.

**Figure 4:**
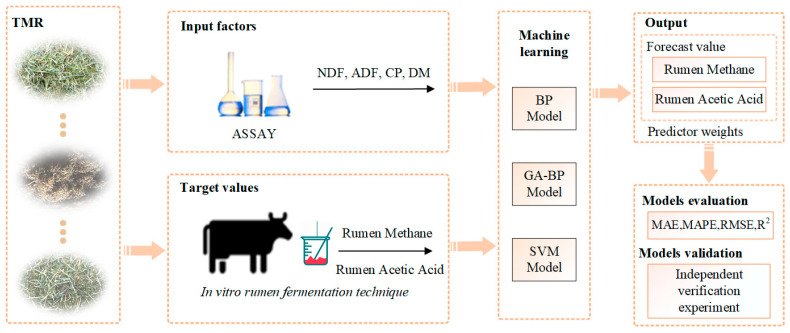
This study’s methodology encompassed four distinct phases: introduction of predictor and target variables; execution of modeling processes (including BP, GA-BP, and SVM models); generation of output results; and implementation of comprehensive evaluation and validation procedures.

However, as there is no definitive criterion for justifying the evaluation [14], it becomes imperative to further validate the capability of rumen models in simulating various types of rumen fermentation and methane production in vitro. Therefore, an independent verification experiment was conducted in Changchun, China, in 2021 to test the models’ predictions and investigate the applicability of in vitro rumen models. The methodology of this study consisted of four distinct phases. A simplified workflow chart (Figure 4) depicting the testing process is presented below.

## 3. Results and Discussion

### 3.1. Model Evaluation

**Figure 5:**
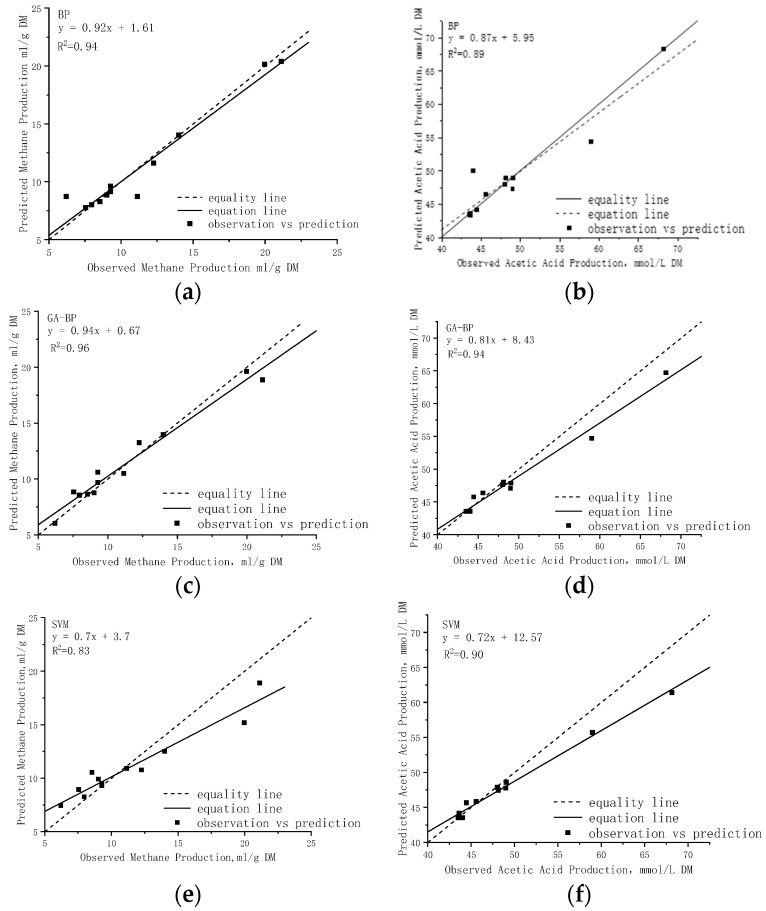
Regression results of rumen methane and rumen acetic acid in different models. (**a**) rumen methane BP−model; (**b**) rumen acetic acid BP−model; (**c**) rumen methane GA−BP model; (**d**) rumen acetic acid GA−BP model; (**e**) rumen methane SVM model; (**f**) rumen acetic acid SVM model.

In this study, all prediction models (BP neural network model, GA-BP neural network model, and Support Vector Machine model) were trained and tested using the same samples, with implementation carried out on the Python platform. Multiple training and testing iterations were performed for both rumen methane and rumen acetic acid as predictive indicators. Figure 5 presents the regression results of these two indicators for the various models.

**Figure 6:**
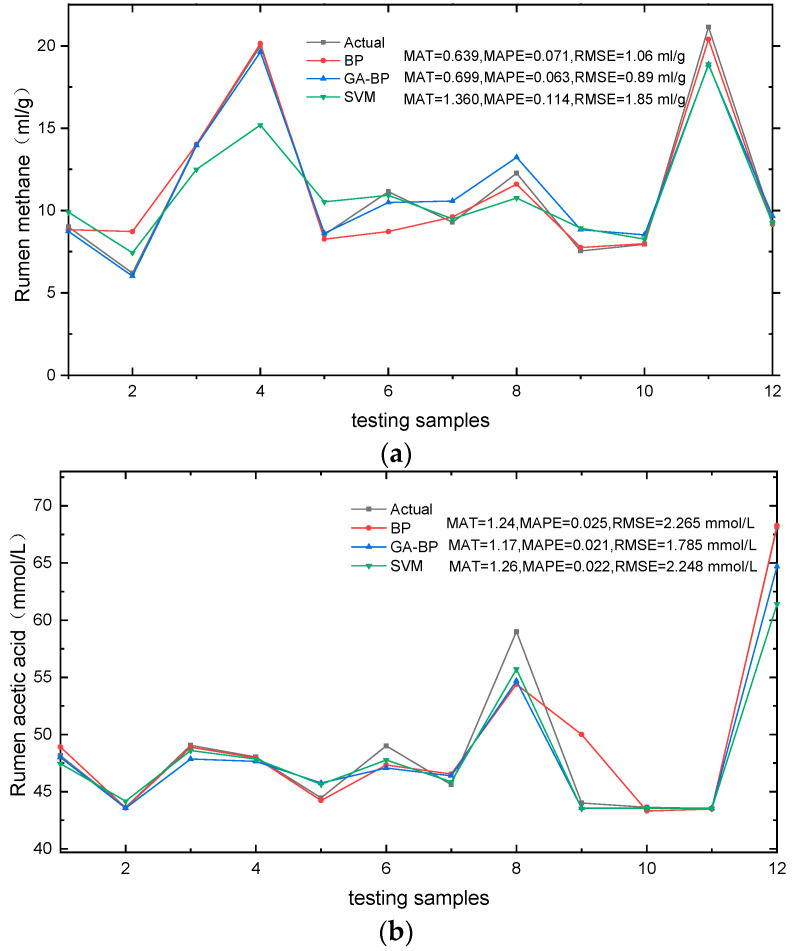
Comparison of simulated and observed rumen methane values. (**a**) Rumen methane models and rumen acetic acid values, (**b**) rumen acetic acid models by different models.

The forecasting accuracy of different models is assessed using three error measures (MAE, MAPE, and RMSE). To mitigate the influence of randomness, each algorithm was executed independently 10 times, and the average prediction error value was computed. Figure 6 illustrates a comparison of the prediction performance for rumen methane and rumen acetic acid by different models.

A comparative analysis of the metrics revealed that all models (in Figure 5) effectively simulated the response patterns of TMR diet composition to cow rumen fermentation. The results showed that the R^2^ values exceeded 0.80 for all models, with the GA-BP model exhibiting the strongest correlation among the testing samples (R^2^ = 0.96 for rumen methane and R^2^ = 0.94 for rumen acetic acid). The forecasting accuracy of these models are presented in Figure 6, where similar prediction errors were observed for ruminal fermentation variables. Specifically, Figure 6a illustrates the simulation of rumen methane across twelve diets, ranging from 3.03 mL/g to 20.39 mL/g, while measured values ranged from 6.2 mL/g to 21.13 mL/g, the average RMSE of the three models was 1.33 mL/g, with the GA-BP model exhibiting a reduction in RMSE values by 16.03% and 51.89% compared to the BP model and SVM model, respectively. Notably, sample NO.11 exhibited a maximum simulated methane value of 20.39 mL/g followed by sample NO.4, indicating a lower energy utilization efficiency in diets with higher simulated methane levels—a finding consistent with experimental results in dairy cow production. The results presented in Figure 6b demonstrate the efficiency of the models’ predictions for rumen acetic acid, as indicated by the low average MAPE (ranging from 0.021 to 0.025) and RMSE values within the range of 1.785 mmol/L to 2.248 mmol/L. The literature and previous studies have demonstrated a positive correlation between the rumen acetic acid content in rumen fermentation and the carbohydrate content in the TMR diet. Notably, the testing sample NO.12 exhibited the highest simulated value of rumen acetic acid content, reaching 68.15 mmol/L. Additionally, TMR diet sample NO.12 demonstrated the highest content of structural carbohydrate NDF and ADL among all samples. Importantly, these simulation results are consistent with the findings of previous studies.

The comparative analysis (in Figure 6) reveals that the lowest mean absolute error (MAT) does not consistently correspond to the same model. However, it is noteworthy that the GA-BP model consistently exhibits the lowest root mean square error (RMSE). These slight deviations can be attributed to variations in calculation methods, as each approach emphasizes specific aspects of goodness of fit [26]. From the MAE, MAPE, RMSE, and R^2^ metrics, it can be seen that the GA-BP model demonstrates superior performance in testing samples. However, it should be noted that the training examples and test examples of the current prediction models are from the same experiment conducted in 2018. Further studies are required to validate these models.

### 3.2. Model Validation

Research has established that the concentrate-to-forage (C:F) ratio, a key measure of feed composition, significantly influences the type of rumen fermentation, thereby impacting methane production in the rumen [24,32]. Through validation experiments, the constructed model was validated to predict rumen fermentation parameters (rumen methane production and rumen acetic acid), thereby verifying the feasibility and potential for widespread adoption of the in vitro rumen prediction model. The observed values and the simulated values by different models are presented in Table 3.

In the validation experiment, T_2_-sim exhibited a lower methane production than T_1_-sim. Previous research by Sun et al. [27] demonstrated that altering the C:F ratio of the diet can influence rumen methane production, with high-concentrate diets leading to reduced emissions from ruminants, which was consistent with the results of this study. Additionally, Li et al. [11] found that the C:F ratio of the diet significantly affects volatile fatty acids (VFAs) in the rumen, thereby determining rumen fermentation types. Notably, T_2_ has a higher concentrate content, providing more substrate for rumen fermentation and consequently increasing rumen acetic acid levels. Moreover, it should be noted that the dietary structure of T_2_ is more scientifically justified. Our rumen acetic acid model also simulated the same conclusion.

Comparing the results (Table 3) clearly indicates that the GA-BP model was the most effective in vitro model for the verification test, which is consistent with our current findings and aligns with the present results. However, it should be noted that all models slightly underestimated methane predictions, and the discrepancy observed may be ascribed to the variations in methane collection methodologies. During the validation phase, methane production was measured using a real-time in vitro fermentation system, in contrast to earlier modeling that was based on manually gathered data, which could have been incomplete. Therefore, further studies are required to enhance the data source of the model in order to improve its generalization ability and accuracy as an in vitro rumen model.

## 4. Conclusions

In this study, we propose a novel approach that combines machine learning algorithms with in vitro fermentation technology to accurately predict the levels of methane and rumen acetic acid in the rumen of dairy cows fed different diets. The models developed in this study exhibited commendable efficacy. Notably, the GA-BP model outperformed the others, achieving superior predictive accuracy. This model exhibited a significant reduction in RMSE, decreasing by 16.2% and 51.2% compared to the BP and SVM models, respectively, when predicting methane levels. Additionally, for rumen acetic acid prediction, it showed a marked improvement, with a decrease in RMSE by 63.3% and 64.5% relative to the BP and SVM models, respectively.

This study also demonstrates the models’ ability to generalize through validation experiments, as they efficiently predicted outcomes even when significant trial factors were altered such as diet composition, crude-to-concentrate ratio, rumen fluid, methane collection method, etc. Therefore, this approach can serve as an alternative method to in vitro fermentation for quantitatively studying rumen fermentation products and providing guidance for optimizing diet structure, rapidly screening methane-reducing feed options, and improving feeding efficiency.

## Figures and Tables

**Table 1:** Testing samples (10% of data were randomly selected from the sample dataset).

Index	NDF %	ADF %	CP %	DM %	Methane mL/g	Acetic Acid mmol/L
1	35.22	15.19	15.44	37.84	9.02	48.15
2	36.67	18.03	12.92	36.31	6.2	43.61
3	35.2	16.83	14.65	35.6	14	49.05
4	38.05	22.67	15.64	43.72	19.98	55.57
5	34.3	18.43	11.58	48.87	8.54	44.45
6	35.59	17.7	14.53	49.4	11.15	49
7	33.43	18.64	15.6	51.44	9.28	45.6
8	32.67	19.9	15.43	49.78	12.26	59
9	39.65	18.34	12.08	41.19	7.55	44.01
10	36.15	17.39	13.57	45.02	7.96	43.62
11	34.43	17.09	16.23	46.98	21.13	43.52
12	42.93	20.78	12.55	46.58	9.28	68.15

**Table 2:** Nutrient composition of TMR with different ratio of concentrate to roughage (DM basis).

Composition	T_1_ Ingredient, %	T_2_ Ingredient, %
DM (dry matter)	47.26	47.35
CP (crude protein)	16.75	17.62
NPN (non-protein nitrogen)	7.23	9.41
SP (soluble protein)	7.92	10.74
NDICP (neutral detergent insoluble crude protein)	3.92	3.55
ADICP (acid detergent insoluble crude protein)	0.88	0.80
EE (ether extract)	3.45	4.53
Ash	8.12	8.08
NDF (neutral detergent fiber)	41.24	33.61
ADF (acid detergent fiber)	27.52	20.03
Lignin	10.84	8.56
Starch	20.26	24.86
NFC (non-fibrous carbohydrate)	30.44	36.20
CHO (carbohydrate)	71.67	69.80

T_1_, TMR with 40:60 ratio of concentrate to roughage; T_2_, TMR with 60:40 ratio of concentrate to roughage.

**Table 3:** The comparison between the observed values and the simulated values.

Models	T_1_-Obs	T_1_-Sim	T_1_-Re	T_2_-Obs	T_2_-Sim	T_2_-Re
Rumen methane mL/g						
BP	23.11	16.34	0.292	21.94	14.51	0.339
GA-BP	23.11	20.86	0.097	21.94	16.81	0.234
SVM	23.11	13.93	0.397	21.94	12.78	0.418
Rumen acetic acid mmol/L						
BP	38.16	50.22	0.316	41.55	48.73	0.173
GA-BP	38.16	44.49	0.165	41.55	46.04	0.108
SVM	38.16	48.40	0.269	41.55	48.45	0.167

T_1_, Concentrate–forage ratio of 40:60; T_1_-obs, observation of T_1_; T_1_-sim, simulation of T_1_; T_1_-re, relative error. T_2_, Concentrate–forage ratio of 60:40); T_2_-obs, observation of T_2_; T_1_-sim, simulation of T_2_; T_2_-re, relative error.

## Data Availability

The data presented in this study are available upon request from the corresponding author.
